# Predicting alveolar ventilation heterogeneity in pulmonary fibrosis using a non-uniform polyhedral spring network model

**DOI:** 10.3389/fnetp.2023.1124223

**Published:** 2023-02-01

**Authors:** Joseph K. Hall, Jason H. T. Bates, Dylan T. Casey, Erzsébet Bartolák-Suki, Kenneth R. Lutchen, Béla Suki

**Affiliations:** ^1^ Department of Biomedical Engineering, Boston University, Boston, MA, United States; ^2^ Department of Medicine, University of Vermont, Burlington, VT, United States; ^3^ Complex Systems Center, University of Vermont, Burlington, VT, United States

**Keywords:** random networks, isotropy, stiffness, bulk modulus, force transmission, percolation

## Abstract

Pulmonary Fibrosis (PF) is a deadly disease that has limited treatment options and is caused by excessive deposition and cross-linking of collagen leading to stiffening of the lung parenchyma. The link between lung structure and function in PF remains poorly understood, although its spatially heterogeneous nature has important implications for alveolar ventilation. Computational models of lung parenchyma utilize uniform arrays of space-filling shapes to represent individual alveoli, but have inherent anisotropy, whereas actual lung tissue is isotropic on average. We developed a novel Voronoi-based 3D spring network model of the lung parenchyma, the Amorphous Network, that exhibits more 2D and 3D similarity to lung geometry than regular polyhedral networks. In contrast to regular networks that show anisotropic force transmission, the structural randomness in the Amorphous Network dissipates this anisotropy with important implications for mechanotransduction. We then added agents to the network that were allowed to carry out a random walk to mimic the migratory behavior of fibroblasts. To model progressive fibrosis, agents were moved around the network and increased the stiffness of springs along their path. Agents migrated at various path lengths until a certain percentage of the network was stiffened. Alveolar ventilation heterogeneity increased with both percent of the network stiffened, and walk length of the agents, until the percolation threshold was reached. The bulk modulus of the network also increased with both percent of network stiffened and path length. This model thus represents a step forward in the creation of physiologically accurate computational models of lung tissue disease.

## Introduction

Pulmonary disease is a leading cause of death (Li et al., 2020) and has been brought to the attention of the world at large due to the COVID-19 Pandemic (Dorjee et al., 2020). The SARS-CoV-2 virus presents with a variety of symptoms, some of which may be chronic, such as pulmonary fibrosis (PF) (Hama Amin et al., 2022). While PF can have a variety of causes, its treatment options remain limited, and its most insidious form, idiopathic pulmonary fibrosis (IPF), is deadly (Wolters et al., 2014). In general, PF is characterized by stiffening of the lung parenchyma as a result of excessive collagen deposition (Wilson and Wynn, 2009) and cross-linking (Reiser et al., 1986) due to mechanisms that are still not well understood (Wolters et al., 2014). The link between lung structure and function in PF also remains poorly understood, although its spatially heterogeneous nature clearly has important implications for alveolar ventilation. Current diagnostic methods do not have the resolution needed to fully elucidate these implications. However, computational modeling can play an important role in linking structure to function at the alveolar level in the fibrotic lung.

Modern computational models, including spring networks and agent-based models, have provided a useful framework for understanding the effects of disease progression on the mechanical properties of tissue. These models have shown, for example, how self-healing mechanisms (Suki et al., 2020) and cell migration mimicked by random walks (Wellman et al., 2018) can potentially contribute to the progression and repair of fibrotic disease in the lung parenchyma. Typically, however, models of lung parenchyma utilize uniform arrays of space-filling shapes such as squares and/or hexagons in two dimensions (2D) or cubes and truncated octahedrons in three dimensions (3D) to represent individual alveoli (Denny and Schroter, 1995; Maksym et al., 1998; Cavalcante et al., 2005; Denny and Schroter, 2006; Bates et al., 2007; Bates and Suki, 2008; Parameswaran et al., 2011; Oliveira et al., 2014; Wellman et al., 2018; Suki et al., 2020). These arrays have inherent anisotropy due to the limited rotational symmetries of their alveolar units, whereas actual lung tissue is essentially isotropic even in the normal lung (Weed et al., 2015). The mechanical consequences of such anisotropies have been studied (Ma et al., 2013) but are still not fully understood. Furthermore, models based on regular repeating unit structures cannot account for the natural variability in alveolar size that the lung displays (Parameswaran et al., 2009).

A potential solution to the limitation imposed by geometric regularity is to use Voronoi tessellation to generate random assemblies of space-filling units. This has been employed in 3D finite-element models to capture the mechanical heterogeneity of foams (Redenbach et al., 2012). Voronoi tessellation has also recently been used to model lung tissue (Beltrán et al., 2022). Nevertheless, its uses as a realistic representation of lung tissue, and specifically in modeling disease progression remain to be validated. To address these needs, we developed a novel Voronoi-based 3D spring network model of the lung parenchyma, which we refer to as the Amorphous Network. In the present study, we compare and contrast this model to both regular polyhedral network models of the lung parenchyma and to actual lung tissue. We also impose a random-walk agent-based process on the Amorphous Network model in order to simulate the effects of cell movement on the progression of fibrotic disease, and we explore its consequences for lung function.

## Methods

### Creation of the 3D voronoi network

Voronoi geometries are based on distributions of points that define the locations of individual network cells (Okabe, 2000). This allows creation of many uniform space-filling geometries. When the points are arranged in a uniform pattern, cubes, truncated octahedrons, and rhombic dodecahedrons can be created. With randomly placed points, non-uniform shapes fill the space. These shapes all consist of convex polyhedra (Canny and Donald, 1988) with faces composed of triangles. The truncated octahedron, for example, has squares and hexagons as faces, and each face is subdivided into triangular segments that collectively define the various alveolar walls.

In preliminary studies, we found that using a purely random arrangement of points in 3D does not recreate a realistic geometry for the alveolar structure of the lung because some of the points, just by chance, can be very close to each other, which gives rise to distorted alveoli. To avoid this problem, we employed a Poisson Disk Sampling (PDS) (Cook, 1986) approach in which points are initially generated at random and then iteratively accepted if they are more than a minimum distance d from any other point (Bridson, 2007). However, the value of d determines how many alveoli can fit into a space of volume V ; a large d corresponds to a small number of large alveoli, and *vice versa*. Thus, in order to create a model having a given number of alveoli, p , we need to know the corresponding value of d . This can be calculated by considering the volumetric packing efficiency, e , defined by the fraction of space occupied by spheres of radius r=d/2 centered on each point in the PDS distribution such that none of the spheres overlap. In the case of spheres of equal size, we have

$$V_{spheres}=p\frac{4\pi r^{3}}{3}$$ (1)

and e is the ratio

$$e=\frac{V_{Spheres}}{V}$$ (2)

which for a face-centered cubic lattice is approximately 74% (Hales et al., 2017). This represents the largest number of spheres that can be packed into V for a given value of d . Rearranging Eq. 2 gives

$$d=2{\left(\frac{3eV}{4\pi p}\right)}^{\frac{1}{3}}$$ (3)

for a collection of spheres of equal size randomly packed into V, e≅64% (Scot and Kilgour, 1969), which represents the upper bound for the PDS approach. In other words, the hypothetical maximum value of d possible for p points with the PDS approach is obtained by using e=0.64 in Eq. 3:

$$d_{\max}≅2{\left(\frac{0.48V}{\pi p}\right)}^{\frac{1}{3}}$$ (4)

Once a set of points with a specified value of d is generated, a set of non-uniform space-filling polyhedra, the Amorphous Network, is created using the 3D Voronoi algorithm (Okabe, 2000).

### Creation of spring models

The 3D Voronoi model consists of a set of space-filling convex polyhedra (Canny and Donald, 1988) with faces segmented into triangular sections. The mechanical properties of the model are provided by having all the edges of the triangular faces be Hookean springs. The intersections of these springs are the nodes of the network. Prestress was imposed by setting the resting length of each spring to 50% of its length in the Voronoi network. The spring constants were inversely proportional to the resting spring lengths, corresponding to uniform elastic material properties. However, some springs had extremely short lengths and so had correspondingly high stiffnesses. This tended to make the determination of the global equilibrium configuration of the network numerically unstable, so we imposed a minimum spring length.

In order to allow for stiffer regions of tissue to contract without distorting the shape of the entire tissue, the boundary nodes on the faces of the tissue were allowed to move within the plane of their respective faces.

### Agent-based modeling of pulmonary fibrosis

Based on our previous agent-based modeling work in 2D (Oliveira et al., 2014), agents representing migrating fibroblast cells were placed at random nodes within the network and then allowed to take a random walk of n steps between adjacent nodes, with n taking values of 1, 10, and 100. After n steps, the agents were moved to a new random node where they undertook a new random walk. Springs that were walked over were stiffened by a factor of 10, since experiments have shown that fibrotic tissue is 2–30 times stiffer than normal (Liu et al., 2010; Liu and Tschumperlin, 2011) (note that this is less than the stiffening factor of 100 that was used in a previous modeling study Oliveira et al., 2014). A spring was only allowed to be stiffened once, so successive walks across it did not lead to further stiffening. This process was continued until a specified percentage, P , of all springs had been stiffened. Varying P from 1% to 50% simulated increasing stages of disease progression. Using n=1 resulted in spatially random spring stiffening throughout the network, while n>1 led to increasing degrees of connectedness between the stiffened springs. Each value of *p* and n was tested in triplicate, each time with different realizations of random agent movement and different Amorphous Networks.

As P increases, percolation occurs at the percolation threshold when a contiguous chain of stiffened springs spans the network, or percolates across it, for the first time (Stauffer and Aharony, 1992). Such a percolation is expected to have a strong effect on the bulk modulus of the system (Oliveira et al., 2014). The percolation threshold, $p_{c}$ , for irregular 3D networks can be estimated as (Zhukov et al., 2019)

$$\ln p_{c}\left(x\right)=\frac{5.04}{x}-2.06$$ (5)

where x is the node degree (average number of links per node, where springs are the equivalent of links in this network). We used this equation to estimate the percolation threshold for our network.

### Geometric validation

To validate the structure of our Amorphous Networks, we compared their 2D and 3D geometries to cubic and truncated octahedral network and lung structural data from the literature. For the 2D validation, slices were taken through the 3D network at 15-degree rotation intervals between 0 and 165° relative to one of the principal axes of the network. This was chosen due to the rotational symmetry in the truncated octahedral network. We determined the distribution of cross-sectional areas of the alveoli in the slices. Areas that were less than 10% of the mean area for a given slice were removed. The angles between alveolar walls at each intersection were estimated by approximating each wall as a straight line between connected intersections and measuring the angles between these lines. These distributions were compared to measurements taken from lung slices by Oldmixon et al., 1988, which only included 3 wall intersections, so to make the comparisons equivalent, we considered only 3-wall intersections in the Amorphous Network slices. For the 3D validation, we determined the distributions of the polyhedral volumes.

To evaluate the fibrotic changes in the 2D slices, stiffening of the springs was translated to disease progression in the faces. Each alveolar wall is represented by a face that is composed of three springs, and so a face that intersects with the slice was set to a disease progression of 0–3 based on the number of springs that were stiffened. In the 2D image, the walls were then colored and thickened based on the intensity of disease progression to better visualize fibroblastic foci.

### Simulated breathing

To simulate breathing, the volume of the network was doubled by imposing a linear strain of 0.26 onto the nodes of the network. These strains were directed radially outward from the center of the network. We then determined the equilibrium configuration of the network as that which minimized its total elastic energy using a first-order gradient descent method using a custom C++ code (Herrmann, 2021). The ventilation of each of the polyhedra that represented individual alveoli was calculated from the difference between the polyhedral volume before and after the network was stretched according to

$$\epsilon_{V}=\frac{V_{high}-V_{low}}{V_{low}}$$ (6)

where $\epsilon_{V}$ is the volumetric strain, $V_{high}$ is the stretched volume, and $V_{low}$ is the baseline volume. Ventilation heterogeneity was characterized by computing the coefficient of variation (CV) of $\epsilon_{V}$ for each disease state.

To evaluate how the mechanical properties of the network changed as a result of disease progression, the bulk modulus B of the network was calculated from the total volume V of the network and the sum of the elastic energies in all the spring as follows. First, the change in energy, ΔE , of the network when V is increased by ΔV is

$$\Delta E=\frac{1}{2}H\Delta V^{2}$$ (7)

where H is the volumetric modulus. Since H=B/V , Eq. 7 rearranges to become

$$B=\frac{2V\Delta E}{{\Delta V}^{2}}$$ (8)

Equation 8 was used to evaluate the bulk modulus of the networks.

### Force propagation

To evaluate the effects of force transmission due to structural differences between the Amorphous Network and the regular networks, a 2D analysis was conducted on the Amorphous Network compared to a regular hexagonal network. The process of creating the 2D network was similar to the 3D network except that area efficiency was used instead of volumetric efficiency to create the PDS. All springs were set to have a resting length of 0.5 times their length in the Voronoi configuration with an imposed minimum length. A small region in the center of each network, approximately 12.5% the diameter of the total network, was stiffened 10-fold, and the complete spring networks were solved for their equilibrium configuration. The angles of the reaction forces on the fixed boundary nodes of the network were evaluated to test for inherent directionality of forces in the network.

### Computation

Matlab R2021b was used for calculations, statistical analysis, and creation of figures.

## Results

### Geometric analysis

**FIGURE 1:**
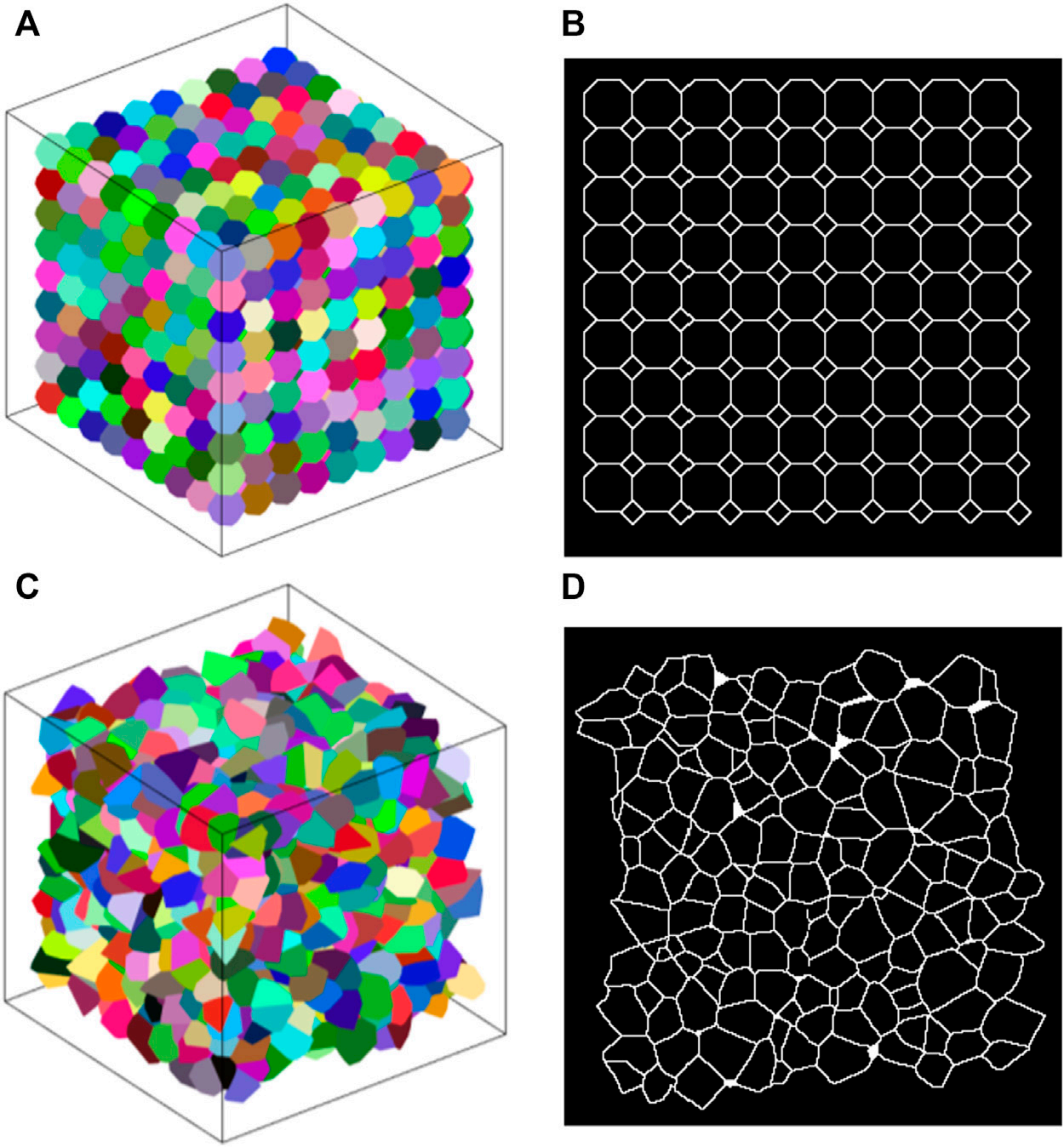
**(A)** Example truncated octahedral network. Each alveolus (different colors) is represented by a truncated octahedron. **(B)** 2D slice through a truncated octahedral network. The pattern of areas is regular and has a bimodal distribution. **(C)** Example amorphous network. Different colors represent individual alveoli. **(D)** Slice of an amorphous network where there is no clear pattern.

The average (±SD) number of polyhedra in the Amorphous Network was 1232 (±12). Each polyhedron representing an alveolus had an average of 73 ± 16 edges and connected to an average of 15 ± 3 surrounding polyhedra. This is similar to the truncated octahedral network in which each truncated octahedron has 14 faces and connects to 14 surrounding octahedra. Furthermore, if the faces of a truncated octahedron are divided into the fewest possible triangles by drawing lines between its nodes (4 triangles per hexagon and 2 triangles per square), the resulting polyhedron has 66 edges. Examples of the truncated octahedral network, Amorphous Network, and their cross-sectional slices are shown in Figure 1.

A slice through the cubic network along one of the primary axes (not shown) comprises a rectangular grid (the aspect ratio of each rectangle depends on the angle of the cut relative to the axes of the cubes). Each node thus has 4 links, and so is denoted as degree 4. The alveolar volumes are all identical with a CV of 0, and there is 90-degree rotational symmetry about each of the principal cube axes. These are characteristics that do not match those of real lung tissue.

**FIGURE 2:**
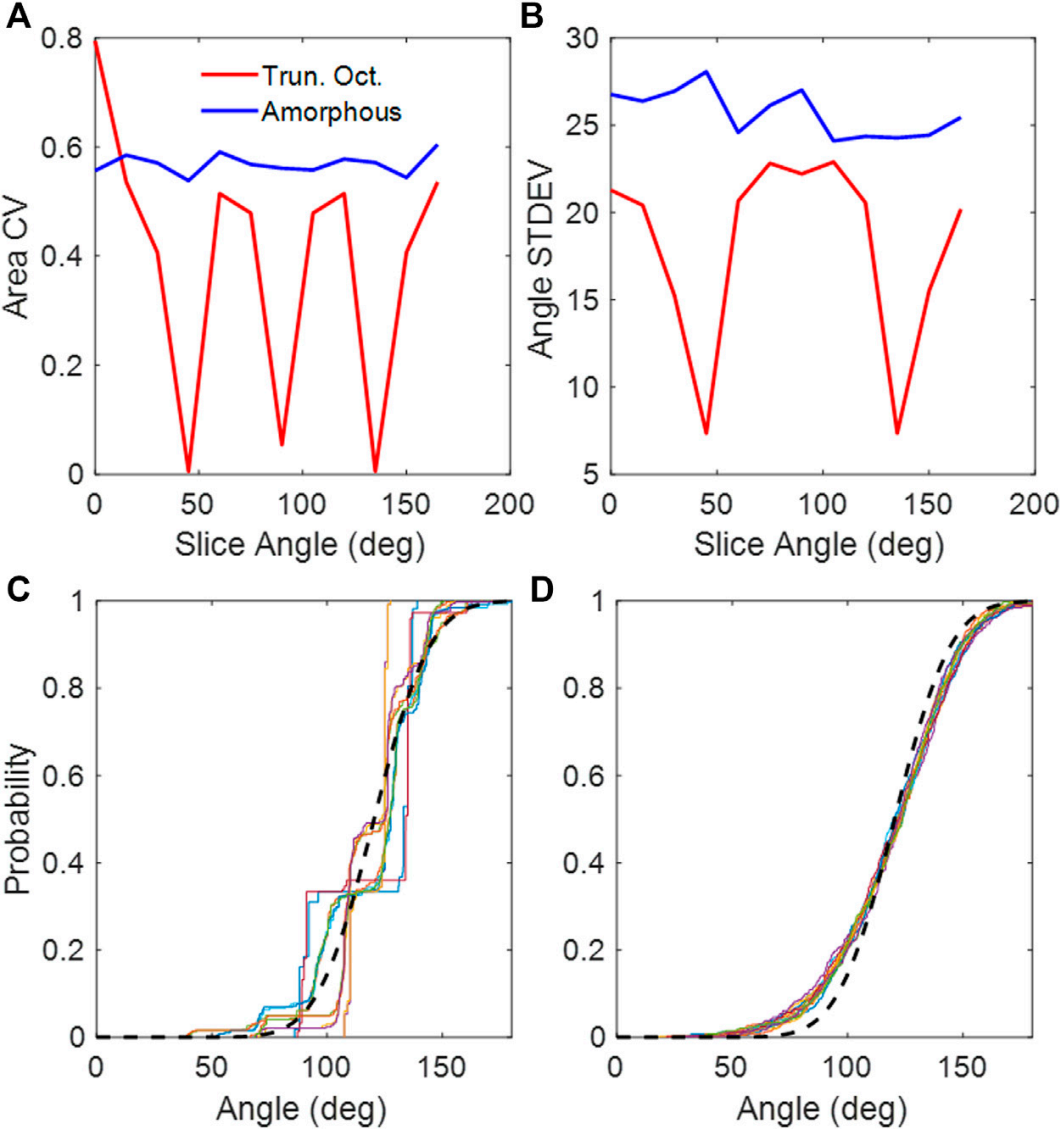
**(A)** CV of areas collected from 2D slices of the truncated octahedral network and the amorphous network. **(B)** Standard deviations of dihedral angles collected from 2D slices of the truncated octahedral network and the amorphous network. **(C)** Cumulative distribution of dihedral wall angles from truncated octahedral network. **(D)** Cumulative distribution of dihedral wall angles from amorphous network. The different colors in **(C,D)** correspond to slice angles from 0 to 165 in 15-degree intervals. The black dashed line is the distribution from published lung data (Oldmixon et al., 1988).

The truncated octahedral network (Figure 1A) is a space-filling geometry that is often used in modeling lung parenchyma but, like the cubic network, it also has limited rotational symmetry. The alveolar areas seen in 2D slices can exhibit regular patterns and can assume either unimodal or bimodal distributions. An example is the slice shown in Figure 1B, which consists of uniform octagons and squares. At cut angles of 45, 90, 135, the CV of the area distribution approaches zero (Figure 2A) as the patterns become uniform hexagons or squares. This is not characteristic of lung tissue. At other slice angles, the SD varies strongly with angle (Figure 2B). The cumulative distributions of dihedral angles Figures 2D and D, show a staircase pattern that is not similar to measured data (Oldmixon et al., 1988). Furthermore, the alveolar volumes in this network are identical, which again is not physiological.

We constructed Amorphous Networks with e values (Eq. 2) of 0% (random network), 20%, and 40%. The dihedral angle distributions obtained with e= 0%; e= 40% were significantly different from each other, with the CV of the alveolar volumes for the e= 40% Amorphous Network showing less heterogeneity than the random network. We found that e = 10% gave a structure reminiscent of real lung tissue. For the healthy Amorphous Network, the CV of the volumes was 0.42 compared to the literature value of 0.63 ± .08 (Parameswaran et al., 2009). The equivalent diameters calculated from the areas of the slices from the Amorphous Network had a CV of 0.32 compared to the literature value of 0.25 ± .05 (Parameswaran et al., 2009). The dihedral angles from the 2D slices had a standard deviation of 23.8° compared to the mean literature value of 19.2° (Oldmixon et al., 1988).

**FIGURE 3:**
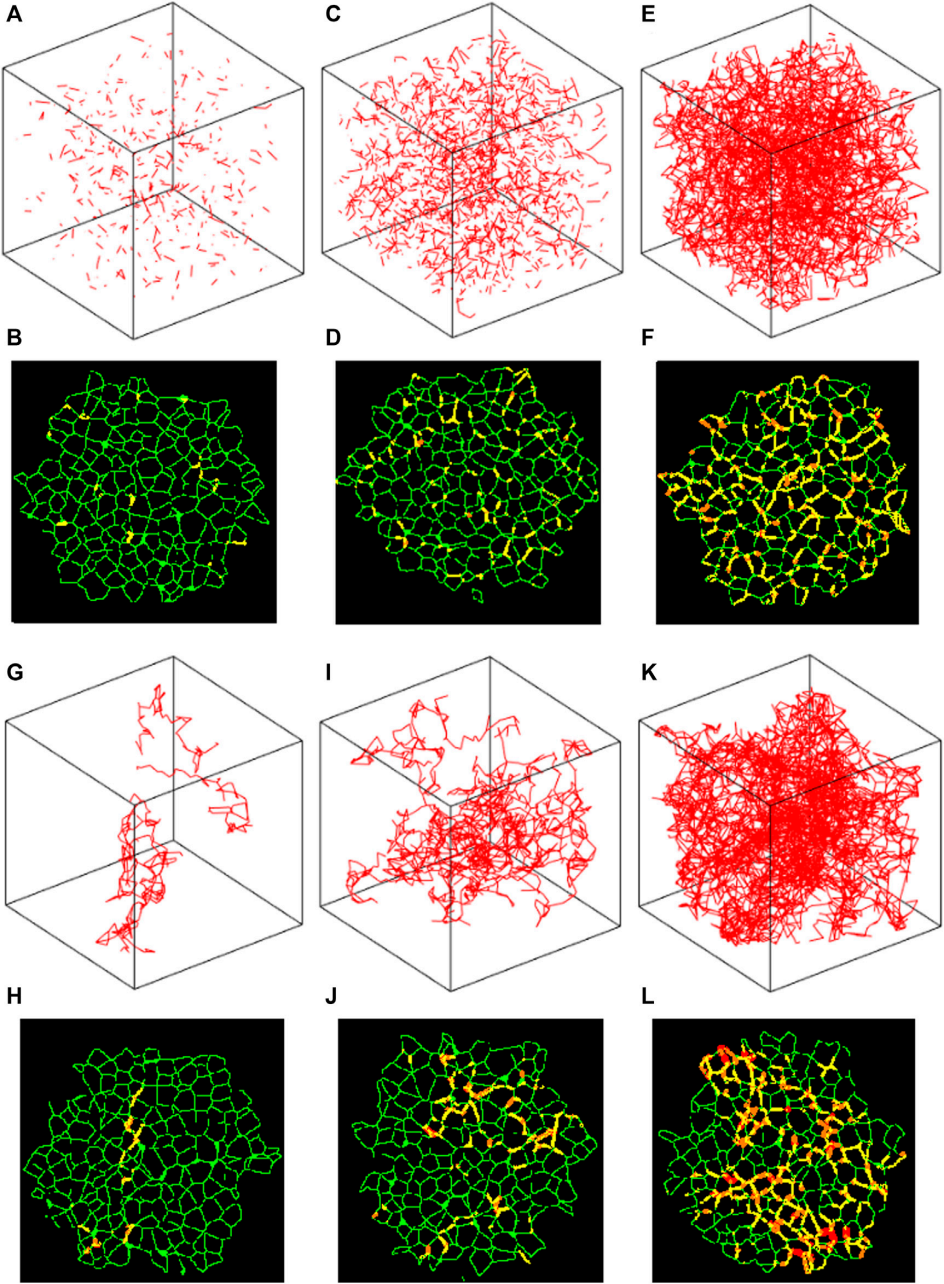
Stiffened springs in the Amorphous Network. **(A,B,G,H)** 1% disease progression. **(C,D,I, and J)** 5% disease progression. **(E,F,K, and L)** 20% disease progression. **(A–F)** Agent step length of 1, i.e., random spring stiffening. **(G–L)** Agent step length of 100, showing clear clustering of disease progression. 2D images **(B,D,F,H,J, and L)** Healthy walls in green, 1/3 progression in yellow, 2/3 in orange, and complete in red.

Figure 3 compares the fibrotic structures both in 2D and 3D generated by the agent-based model for n = 1 and n = 100, each with disease progression percentages of 1%, 5% and 20%. Figures 3A–F demonstrate that with n = 1, fibrosis develops in isolated patches of a few links in size that lead to relatively homogeneous disease. In contrast, when n = 100, fibrosis mostly spreads along continuous paths leading to clumping of the stiffened springs (Figures 3G–L).

**FIGURE 4:**
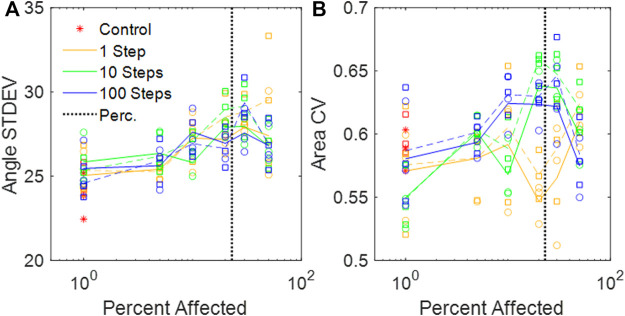
**(A)** Standard deviations of the dihedral wall angles in the amorphous networks vs. percent disease progression. **(B)** CV of areas from amorphous networks vs. percent disease progression. **(A,B)** Circles and solid lines represent values at baseline network volume, and squares and dashed lines represent distributions at stretched volume. The vertical black dotted line is the percolation threshold.

We used e = 10% for the agent-based disease progression model. The average number of springs per node was 8.8, so the estimated percolation threshold using Eq. 5 was approximately 0.23. Across all models, the SD of the dihedral angles increased slightly with increasing disease progression (Figure 4A), and this did not appear to change with the expanded network. There were no clear trends in the CV of the alveolar areas from any of the networks (Figure 4B) apart from an increase in CV in the expanded networks.

### Ventilation heterogeneity

**FIGURE 5:**
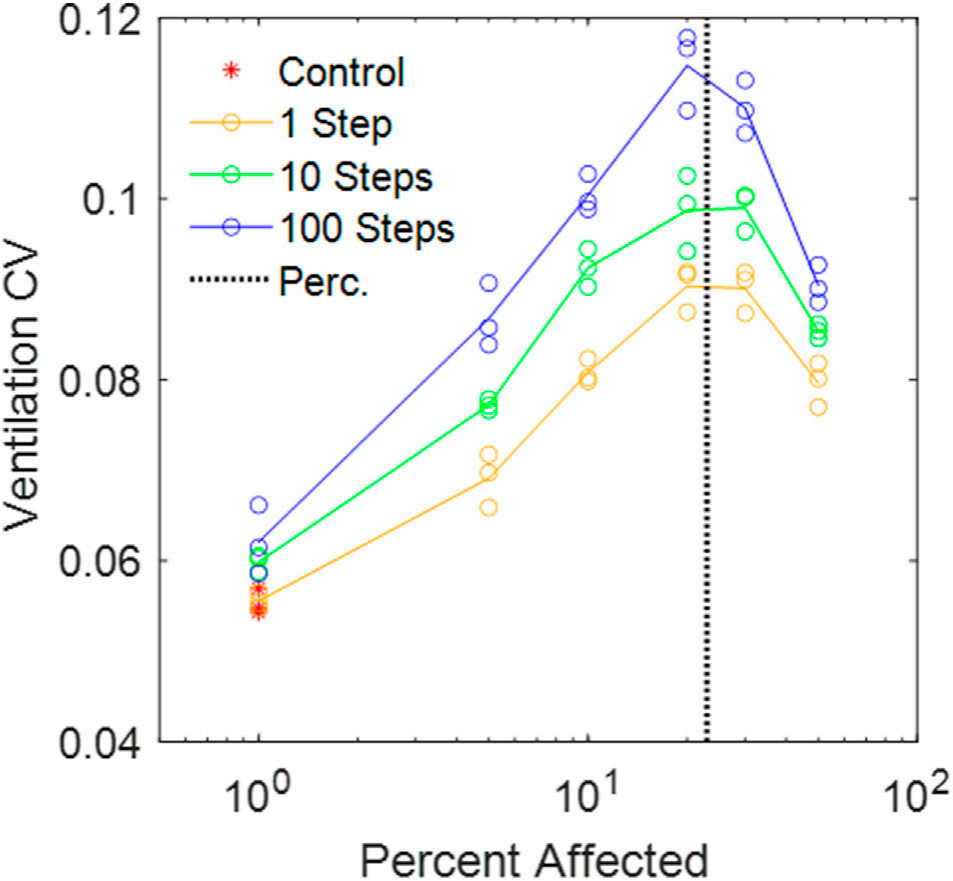
CV of alveolar ventilation in the amorphous network vs. percent disease progression for different random walk lengths. The vertical black dotted line is the percolation threshold.

The ventilation CV increased both as the percent disease progression increased and as n increased, until the percolation threshold was crossed after which the CV dropped (Figure 5). This was consistent for all values of n . The most highly clustered model (that with n=100 ) produced the highest ventilation heterogeneity with a CV reaching close to 12% near the percolation threshold.

The network with the greatest ventilation heterogeneity, the 20% disease progression with a walk length of 100, was tested at 4 network sizes between 200 and 2000. The ventilation CV did not appear to change above ∼500 alveoli (Supplemental Figure S1).

### Bulk modulus

**FIGURE 6:**
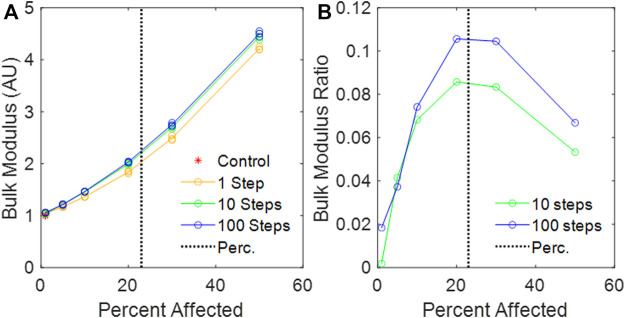
**(A)** Calculated bulk modulus of the amorphous network vs. percent disease progression, normalized to mean control bulk modulus. **(B)** Relative change in bulk modulus between the 1-step and the 10-step and the 1-step and 100-step models vs. percent affected. The vertical black dotted line is the percolation threshold.

The value of B also increased as the percent disease progression increased (Figure 6A). Figure 6B shows the difference between B obtained n=10 and n=100 divided by B obtained with n=1 . Inspection of Figure 6B shows the rate of change of B with n=100 is greater than for the random network, with a maximum difference of over 10% near the percolation threshold.

### Force isotropy

**FIGURE 7:**
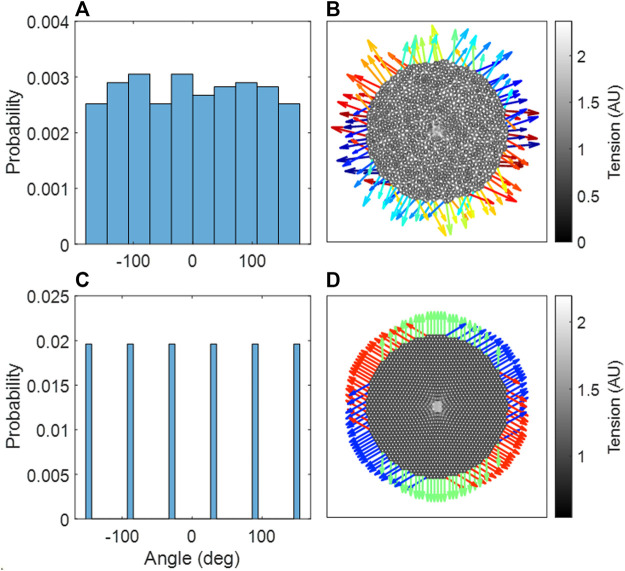
**(A)** Probability distribution of reaction force angles in the amorphous network, an aggregate of three independent network configurations. **(B)** The amorphous network, with stiffened region in center. **(C)** Probability distribution of reaction force angles in the hexagonal network. **(D)** The hexagonal network, with stiffened region in center. **(B,D)** The reaction forces at the boundary are shown as arrows, where the color indicates the angle of the reaction force.

Figures 7A, B demonstrate that the Amorphous Network had no preferential reaction force directions at the boundary. In contrast, the hexagonal network had clear directionality in the reaction forces at the boundary nodes (Figures 7C, D). It is also clear that the Amorphous Network has more heterogeneity in force than the hexagonal network.

## Discussion

We have introduced a novel 3D Voronoi-based spring network, the Amorphous Network, as a more realistic model of the network structure of the lung parenchyma that recapitulates the naturally occurring random variation in alveolar shapes and sizes. We applied an agent-based model simulating fibrosis development to investigate how ventilation heterogeneity and bulk tissue modulus change as disease progresses. Our results show that the novel Amorphous Network provides a more realistic alveolar geometry compared to networks that use uniform polyhedra such as the truncated octahedral network (Figures 1, 2). We have also shown that an increase in the degree of clustering of fibrotic lesions (Figure 3), achieved by allowing longer random walks of fibrotic agents in the network, increases the CV of alveolar ventilation (Figure 5) and leads to a moderate increase in B (Figure 6). Lastly, we demonstrate using 2D simulations in Figure 7 that force propagation is isotropic in the Amorphous Network but not in a regular hexagonal network.

An interesting property of the Amorphous Network is that it is structurally and mechanically isotropic on average despite having a heterogeneous structure. In fact, compared to regular networks that are anisotropic (Figure 2), the isotropy of the Amorphous Network is a consequence of its heterogeneity. This has interesting consequences for force transmission. For example, the mechanical force of local stiffening would be transmitted much farther in a cubic network if one of the axes of the cubes coincided with the direction of an imposed force such as gravity (unpublished observation). Preferential directions of force are also evident in the hexagonal 2D network (Figure 7B). In contrast, our simulations illustrated in Figures 7A, B show no preferential direction for force transmission in the Amorphous Network, consistent with observations in lung tissue (Weed et al., 2015). The Amorphous Network achieves this by dissipating forces in random directions. This has important biologic implications because anisotropy of force transmission could result in mechanotransduction being direction dependent, which is apparently not the case in reality. Randomness may also be a key property of development. It has been shown that a regular bifurcating airway network would amplify the smallest error in structure during growth, as opposed to a heterogenous fractal airway network that is tolerant to such error propagation (West, 1990). The native heterogeneity of alveolar structure may also be similar. For example, a small error in the truncated octahedron may become amplified during growth but this will not happen in the Amorphous Network that builds natural variability into its space-filling structure.

The heterogeneity of the Amorphous Network is also displayed in its cross-sections (Figures 1, 3). The 2D slices from uniform polyhedral networks can display repeated patterns that can form uniform or bimodal area and angle distributions (Figure 2), and therefore they do not recapitulate the geometry reflected in physiologic data. The Amorphous Network, however, does not have geometric preferences at particular angles, and has heterogeneities similar to real parenchyma. On the other hand, the area distributions found with the Amorphous Network in the present study did not change significantly as disease progressed or the value of n increased (Figure 4), both of which would be expected. This is likely due to the fact that we did not allow the most highly stressed alveolar walls in the model to rupture. This would result in the merging of the two alveoli, and a corresponding decrease in surface area and overall material stiffness. Rupture was previously introduced to account for the emergence of subpleural honeycomb structures that produced increased heterogeneity in a previous 2D model (Wellman et al., 2018).

The cross-sections shown in Figure 3 also clearly demonstrate the localizing of fibrotic regions. In the models where the distribution is random, the fibrotic regions are spread across the whole sample, whereas in the models with walk length of 100, the regions of disease and lack of disease are clearly distinguishable from each other. The clusters also appear to have more concentrated fibrosis progression, shown in red, with increased step length compared to the random fibrotic regions. It is worth noting that as the Amorphous Network reaches equilibrium, concavities may form depending on the parameters of the network, and this is a key element that distinguishes the Amorphous Network from a 3D Voronoi network. These concavities are shown for the Amorphous Network in Figures 1, 3.

The ventilation heterogeneity seen in the Amorphous Network model (Figure 5) represents alveolar level ventilation. To our knowledge, this has not been measured experimentally despite the availability of technologies such as micro-CT (Jones et al., 2016). This lack of alveolar-level data makes a direct comparison of model heterogeneity to heterogeneity in the lung difficult. However, the 12% maximum CV appears to be low compared to reported data (Mata et al., 2021). This may be a consequence of modeling the spread of fibrosis using a simple random walk. The random movement is perhaps physiological under normal conditions, but in fibrosis it can be different. Fibroblasts have the tendency to migrate toward regions of stiff exrtacellular matrix (Lo et al., 2000), and on stiff matrixes they can transdifferenciate to myofibroblasts and deposit more ECM making it progressively stiffer (Hinz, 2009). A patchier diffusion-like spreading could be more realistic whereby the migration of the agents is controlled by local feedback such as tissue stiffness itself, and could lead to more region-specific disease progression, such as at the periphery of the lungs (Wellman et al., 2018). When combined with alveolar rupture, the model by Wellman et al., 2018, was able to reproduce the peripheral honeycombing typical of IPF. Heterogeneous structures in future models could be analyzed using stereology to further validate the geometry, and stereology could be applied to estimate alveolar volumetric distributions from histological images. Additionally, direct measurement of volumes using micro-CT scans would be invaluable to the application of the Amorphous Network (Jones et al., 2016). The behavior of the model as a whole could be compared to measurements from Electrical Impedance Tomography (Tomicic and Cornejo, 2019).

With regard to the stiffness of the lung parenchyma, we first note that phenomena at the percolation threshold often show sharp transitions. For example, B has been shown in other models to suddenly increase when the percent disease progression crosses the percolation threshold (Bates and Suki, 2008; Oliveira et al., 2014).; small isolated clusters of stiff springs have relatively little effect on overall tissue stiffness, but when they suddenly connect together in a contiguous chain that spans the tissue, they are able to collaborate in a much more effective way (Stauffer and Aharony, 1992; Oliveira et al., 2014). The Amorphous Network of the present study, however, does not display such a sharp transition (Figure 6A), probably because the network is prestressed and the springs become stiffened by a factor of only 10 as opposed to the factor of 100 used previously (Bates and Suki, 2008; Oliveira et al., 2014). Thus, the sharpness of the transition at the percolation threshold is related to the difference in properties before and after a bond is occupied by the percolation process. Indeed, additional simulations with the Amorphous Network where springs were set to be 1000 times stiffer generated a much steeper increase of the bulk modulus (Supplemental Figure S2). There are also differences in percolation behavior between 2D and 3D networks. In 2D networks, the common structure used to model the parenchyma is a hexagonal pattern for which the percolation threshold is 0.65, implying that a majority of the springs have to become fibrotic before significantly affecting the stiffness of the network. The percolation threshold in our 3D network is only 0.23, which highlights the importance of dimensionality and structure when using models to mimic diseases.

There are a number of limitations that arise from the simplicity of our Amorphous Network model. The use of volumetric efficiency as a means of controlling the parameters of the Amorphous Network (Eqs 1–4), while straightforward, nevertheless did not reproduce real parenchymal distributions exactly. This could have been due to a number of confounding factors, such as the lack of airways, pleura, and external forces such as gravity and variation in pleural pressure. The effect of gravity on the lungs causes gradient that appears as heterogeneity, where the alveoli at the top of the lung are under a greater stress than those at the base of the lung, which leads to ventilation heterogeneity (Prisk, 2014). We also tested a uniaxial expansion and found that for the same change in volume, the ventilation CV increased compared equiaxial expansion, indicating that there is potential for this model to explore different regions of the lungs to evaluate regional heterogeneity in future models. These omissions also potentially affected ventilation distributions. We also represented tissue stiffness using Hookean rather than non-linear springs, and we did not include either tissue viscoelasticity or surface tension, all of which would have greatly complicated the model and made finding its mechanical equilibrium configuration much more challenging numerically. Furthermore, we modeled fibrosis as a single change in intrinsic spring stiffness, with no gradient allowed for disease progression, no breakage nor healing of the springs, and we employed purely random agent movement without the possibility for mediator-driven chemotaxis or a stiffness-driven biased random walk (Wellman et al., 2018). Additionally, our model does not represent the biological mechanisms that lead to individual forms of IPF. These limitations are shared by a number of previous parenchymal modeling studies such as the 2D percolating model presented by Bates and Suki, 2008 that utilized linear springs and binary fibrotic/non-fibrotic springs. On the other hand, these other 2D models excel at showing fibrotic clusters and area heterogeneity, but they are either 2D (Wellman et al., 2018) or lack natural heterogeneity of the lung structure (Parameswaran et al., 2011). Hence, they cannot predict ventilation distribution of the healthy lung nor can they estimate the worsening of ventilation distribution due to disease. In contrast, the Amorphous Network of the present study is able to reveal a link between the nature of disease progression and changes in alveolar ventilation heterogeneity and overall tissue stiffness (Figures 5, 6). We hypothesize that inclusion of complex features, such as viscoelasticity, rupture, and airways would further increase ventilation heterogeneity, which warrant further investigation. Despite these limitations, the Amorphous Network represents a step forward compared to current uniform models, and future extensions with more volumetric data and quantitative analysis will further develop on this foundation.

## Conclusion

We have introduced and investigated a novel structural network model of the lung parenchyma, and by adding agents, we have demonstrated its ability to predict ventilation heterogeneity and parenchymal bulk mechanical properties as pulmonary fibrosis progresses. This model thus represents a step forward in the creation of physiologically accurate computational models of lung tissue disease.

## Data Availability

The original contributions presented in the study are included in the article/Supplementary Material, further inquiries can be directed to the corresponding author.
