# Supplementary material for: Predicting alveolar ventilation heterogeneity in pulmonary fibrosis using a non-uniform polyhedral spring network model
Source: Front Netw Physiol. 2023 Feb 1;3:1124223. doi: 10.3389/fnetp.2023.1124223 (PMC10013074; doi:10.3389/fnetp.2023.1124223)
Supplement: Supplementary file 1 [file DataSheet1.PDF]

Predicting Alveolar Ventilation Heterogeneity in Pulmonary Fibrosis using a Non-Uniform Polyhedral Spring Network Model

Joseph K. Hall[1], Jason Bates[2], Dylan T. Casey[2], Erzsébet Bartolák-Suki[1], Kenneth Lutchen[1], and Béla Suki [1]

1. Department of Biomedical Engineering, Boston University, MA, USA

2. Department of Electrical and Biomedical Engineering, the University of Vermont, VT, USA

Supplemental Figures

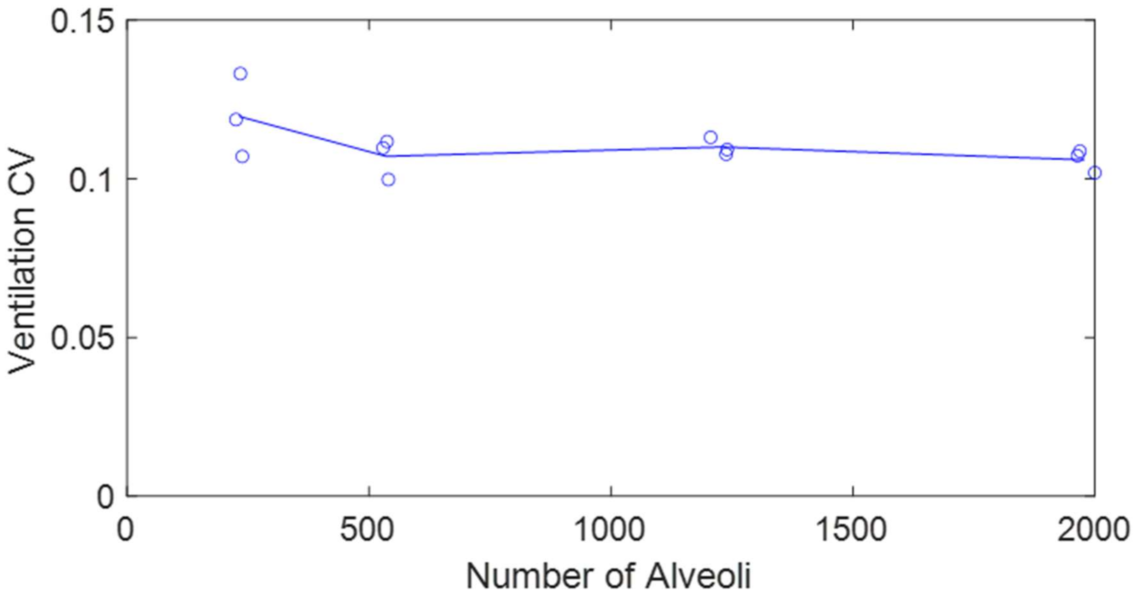

Figure S1: Analysis of ventilation CV vs network size. The network with the highest CV, with 20% spring affected at 100 steps, was evaluated at 4 network sizes in triplicate. Little difference was seen in the ventilation CV, showing that ventilation CV does not depend on the size of a sufficiently large network.

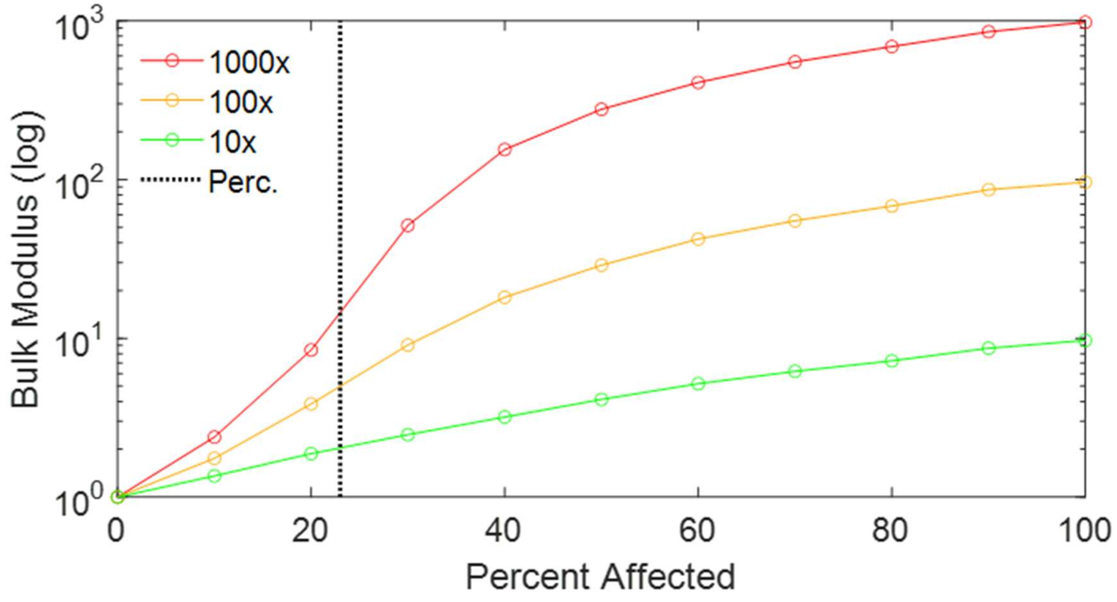

Figure S2: Amorphous Network bulk modulus vs percent network affected with varying stiffening values. Random percolation was modeled by stiffening random springs in an Amorphous Network with around 500 alveoli by 10, 100, and 1000 times stiffness, and then the bulk modulus of the overall network was measured. The percolation threshold of around 23% is most evident in the 1000 times stiffness network. Bulk moduli were normalized by the stiffness of the 0% unaffected network.
